# Assessment of climate-driven variations in malaria incidence in Swaziland: toward malaria elimination

**DOI:** 10.1186/s12936-017-1874-0

**Published:** 2017-06-01

**Authors:** Ting-Wu Chuang, Adam Soble, Nyasatu Ntshalintshali, Nomcebo Mkhonta, Eric Seyama, Steven Mthethwa, Deepa Pindolia, Simon Kunene

**Affiliations:** 1 Department of Molecular Parasitology and Tropical Diseases, School of Medicine, College of Medicine, Taipei Medical University, No. 250, Wuxing St. Sinyi District, Taipei, 100 Taiwan; 2 Clinton Health Access Initiative, Manzini, Swaziland; 3 National Malaria Control Programme, Ministry of Health, Manzini, Swaziland; 4 Swaziland Meteorological Service, Mbabane, Swaziland

**Keywords:** Climate variations, Malaria elimination, Swaziland

## Abstract

**Background:**

Swaziland aims to eliminate malaria by 2020. However, imported cases from neighbouring endemic countries continue to sustain local parasite reservoirs and initiate transmission. As certain weather and climatic conditions may trigger or intensify malaria outbreaks, identification of areas prone to these conditions may aid decision-makers in deploying targeted malaria interventions more effectively.

**Methods:**

Malaria case-surveillance data for Swaziland were provided by Swaziland’s National Malaria Control Programme. Climate data were derived from local weather stations and remote sensing images. Climate parameters and malaria cases between 2001 and 2015 were then analysed using seasonal autoregressive integrated moving average models and distributed lag non-linear models (DLNM).

**Results:**

The incidence of malaria in Swaziland increased between 2005 and 2010, especially in the Lubombo and Hhohho regions. A time-series analysis indicated that warmer temperatures and higher precipitation in the Lubombo and Hhohho administrative regions are conducive to malaria transmission. DLNM showed that the risk of malaria increased in Lubombo when the maximum temperature was above 30 °C or monthly precipitation was above 5 in. In Hhohho, the minimum temperature remaining above 15 °C or precipitation being greater than 10 in. might be associated with malaria transmission.

**Conclusions:**

This study provides a preliminary assessment of the impact of short-term climate variations on malaria transmission in Swaziland. The geographic separation of imported and locally acquired malaria, as well as population behaviour, highlight the varying modes of transmission, part of which may be relevant to climate conditions. Thus, the impact of changing climate conditions should be noted as Swaziland moves toward malaria elimination.

**Electronic supplementary material:**

The online version of this article (doi:10.1186/s12936-017-1874-0) contains supplementary material, which is available to authorized users.

## Background

Thanks to a long-term commitment and successfully deployed malaria control interventions, Swaziland is now aiming to eliminate malaria by 2020. If achieved, it would be the first country in sub-Saharan Africa to meet this ambitious goal [1–3]. Swaziland’s malaria burden is primarily caused by *Plasmodium falciparum*, which is predominately transmitted by the *Anopheles arabiensis* [4]. Swaziland’s combination of confirmatory diagnosis, prompt and efficacious treatment, targeted vector control, health promotion, and active surveillance has been critical in reducing the malaria burden to low levels [1]. With low levels of local transmission, controlling the import of malaria from high-endemic neighbouring countries has become increasingly important [5, 6]. Thus, significant resources have been in place since 2010 to rapidly detect and treat all cases, as well as to investigate people they have been in close contact with, in order to limit additional transmission [5]. The National Malaria Control Programme (NMCP) of Swaziland has been initiating active investigations to follow up all confirmed cases at the household level since 2010. Imported and locally acquired cases can be classified according to travel history, either outside or within Swaziland. Originally, a case was deemed imported if the patient reported having traveled within the past 2 weeks, although this was later increased to 4 weeks in 2012 and to 8 weeks in 2013. Patients who did not report having traveled are deemed to have acquired malaria locally [7].

Since climatic conditions drive parasite and mosquito development, feeding frequency, and disease transmission, short-term climate variations (e.g., temperature, precipitation, and humidity) or irregular climatic phenomena (e.g., El Niño Southern Oscillation) may also be important factors in malaria transmission and the success of elimination programmes in previously unforeseen ways [8, 9]. Multiple studies have been conducted in malaria-endemic areas to investigate the association between climatic variations and malaria epidemics that might be associated with recent climate change [10–12]. For instance, regional climatic indices such as the Indian Ocean Dipole (IOD) or the El Niño Southern Oscillation (ENSO) have been linked to malaria transmission in Kenya, Ethiopia, and South Africa [13–17]. However, the impact of climate conditions on malaria transmission in Swaziland is poorly documented. Using the random forest regression tree approach to generate malaria risk maps of Swaziland in 2011 based on various environmental variables, a study has shown that warmer temperatures, lower rainfall, lower elevation, and close proximity to water contribute to a higher risk of malaria during high- and low-transmission seasons [18]. However, the study only evaluated the environmental influences over a very short period of time. Furthermore, it is possible that certain areas in Swaziland are more vulnerable than others to climate conditions that promote local transmission. Indeed, climatic conditions vary widely in Swaziland despite its relatively small size, and range from humid and temperate in the Highveld region to semi-arid and warm in the Lowveld region [1]. Hence, an analysis of climate conditions and their impact on transmission risk in Swaziland is necessary to reinforce long-term efforts to eliminate malaria and to support the establishment of a malaria early warning system in outbreak-prone Swaziland. The identification of areas or populations at risk of transmission due to climate variations could also enable the delivery of timely control interventions. Therefore, the specific aims of this study were to assess the impact of climatic variations on malaria transmission, and identify specific areas vulnerable to climate conditions that promote transmission in Swaziland.

## Methods

### Malaria incidence

Malaria case and population data provided by the National Malaria Control Programme (NMCP) for 1985–2015 show that annual malaria incidence sharply decreased after 1995 following the successful application of control measures (Additional file 1).

The key intervention strategy in Swaziland is integrated vector management (IVM), which combines both indoor residual spraying (IRS) and long-lasting insecticidal nets (LLIN) to interrupt malaria transmission [1, 19]. Artemisinin-based combination therapy (ACT) recommended by the WHO is used for treating malaria patients because a high level of chloroquine resistance has been found in South Africa and Mozambique [20]. Mefloquine is recommended as a prophylaxis for people traveling to malaria-endemic areas [1]. Monthly incidence data for Swaziland’s four major administrative regions of Hhohho, Manzini, Lubombo, and Shiselweni are available after 2000. Hence, the monthly incidence data in Swaziland from 2001 to 2015 were used to evaluate the influence of climate variations. The geographic clusters of imported and locally acquired cases between 2010 and 2015 are shown in Additional file 2 for reference.

### Climate data

**Fig. 1:**
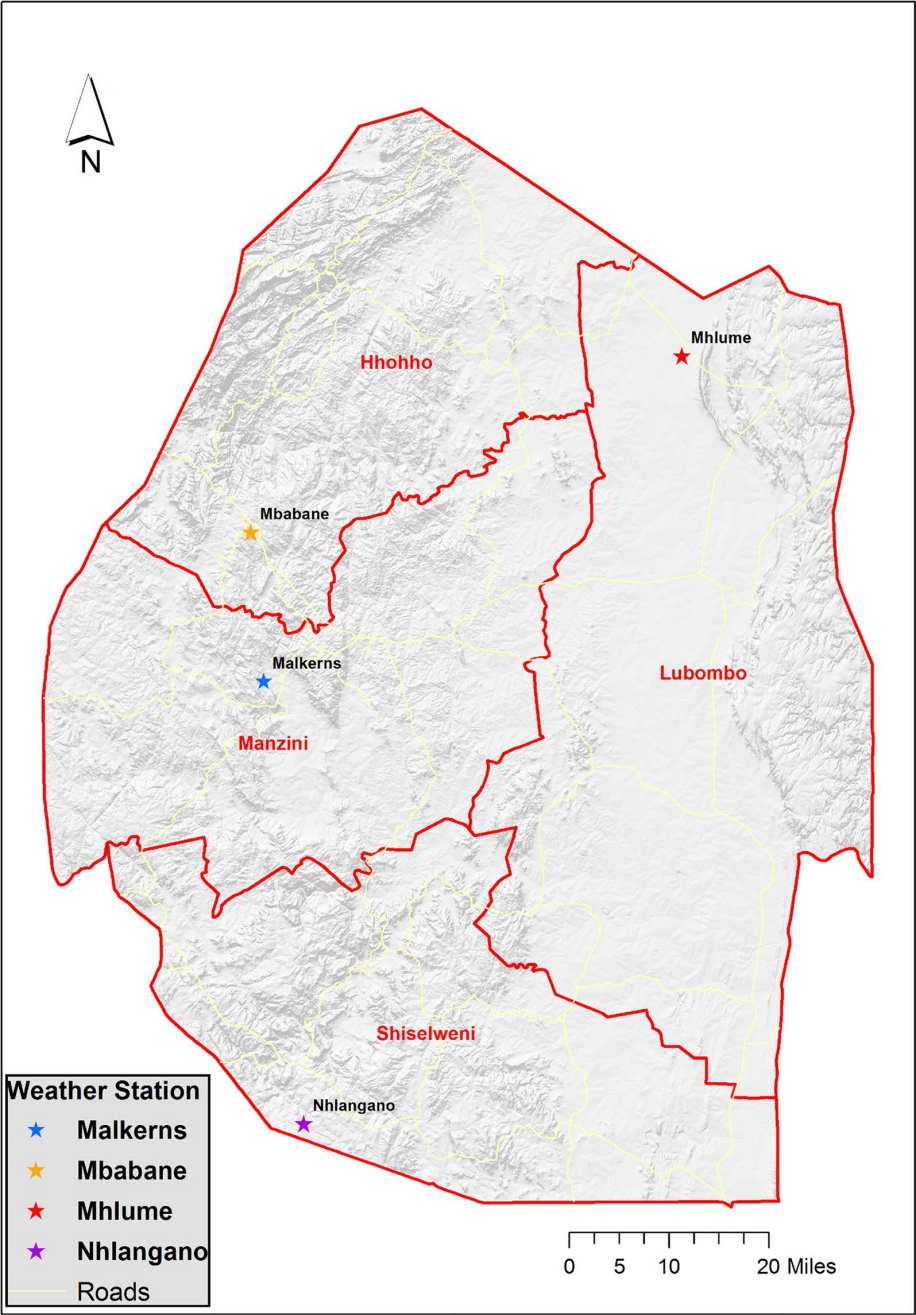
Locations of weather stations in the four major administrative regions in Swaziland (the base map* highlights* the elevation differences in Swaziland: the Highveld is in the west and the Lowveld is in the east)

Meteorological data, including maximum and minimum temperature and precipitation, from 1985 to 2015 are available from 14 weather stations within Swaziland. However, not all stations collected complete data throughout the study period, so the climatic data were mainly obtained from one station that has as much data as possible in each of the four major administrative regions (Fig. 1). Monthly climatic variables were summarized from daily maximum temperature, daily minimum temperature, and daily precipitation (inches). To handle missing data, proxy environmental parameters were derived from satellite remote sensing images using EASTWeb software [21]. Daytime and nighttime land surface temperatures (LST) were derived from the Moderate Resolution Imaging Spectroradiometer (MODIS) MOD11A2 product, and precipitation was derived from Tropical Rainfall Measuring Mission (TRMM) microwave satellite images (3B42 Version 7 product). Although land surface temperature and air temperature are not the same measurements, these variables are strongly correlated [21, 22]. Pearson correlation coefficients, ranging from 0.72 to 0.92, indicated a strong correlation between weather station and remote sensing data (Additional file 3). Finally, linear regression models were constructed to interpolate missing values, and the predicted climatic parameter values were used in the analysis.

Regional climate phenomena were also considered in the analysis. The multivariate El Niño Southern Oscillation Index (MEI), which is an integration of six atmospheric variables, was selected to evaluate the influence of irregular climate variations. Positive values indicate a warm phase (El Niño) and negative values indicate a cold phase (La Niña) [23]. The MEI is available from the Earth System Research Laboratory, National Oceanic and Atmospheric Administration (NOAA) (http://www.esrl.noaa.gov). Malaria and climate data were processed in SAS 9.4 (SAS Institute Inc. Cary, NC, USA).

### Time-series analysis

Monthly malaria incidence and meteorological variables from 2001 to 2015 were analyzed using a seasonal autoregressive integrated moving average (SARIMA) model. In this analysis, time-series data with regular seasonality were decomposed into seasonal and non-seasonal components. Differencing was applied to remove non-stationarity. The simplified notation for SARIMA is

$$ARIMA\left( {p, \, d, \, q} \right) \times \left( {P, \, D, \, Q} \right)S$$

where *p* indicates the non-seasonal autoregressive (AR) order, *d* is non-seasonal differencing, and *q* indicates the non-seasonal moving average (MA) order. *P*, *D*, and *Q* are the corresponding seasonal components. *S* indicates the period, which in this case is 12 months. The importance of climate variables was assessed by the Akaike Information Criterion (AIC), where a smaller AIC indicates better model performance [24].

Non-linear relationships between climate conditions and mosquito ecology have been reported previously [25–27]. In areas where malaria risk was found to be associated with monthly climate variations in the time-series model, we used distributed lag non-linear models (DLNM) to investigate non-linear relationships between climate factors and malaria transmission. Multicollinearity in lagged effects and non-linear relationships can be handled by using the bi-dimensional function [28–30]. The model is expressed as

$$Y_{t} = quasi\; Poisson\,(t = 1,2,3 \ldots ,n)$$

$$\log \left( {\mu_{t} } \right) = \alpha + \sum\limits_{h = 1}^{H} {\beta_{k} (X_{t,h} ) + \log \left( {Pop} \right) + s\left( {month, \rho } \right) + year + \varepsilon_{t} }$$

under a quasi-Poisson assumption to overcome overdispersion. *Y*_*t*_ indicates the monthly number of malaria cases, while log(*μ*_*t*_) is the expected monthly malaria incidence, and *X*_*t,h*_ indicates climatic variables at month *t* and lag month *h*. *H* is the maximum lag (12), *Pop* is an offset to control for the underlying population, and *α*, *β*_*k*_, and *ε*_*t*_ are the intercept, coefficients of covariates, and error term respectively. To control for seasonality, year and smoothed month with degree of freedom (*ρ* = 6) were also included. The natural cubic smooth function was applied to maximum temperature, minimum temperature, precipitation, and the multivariate El Niño Southern Oscillation Index. SARIMA and DLNM analysis were carried out in R 3.2.5 using the packages *dlnm* and *forecast*.

## Results

**Fig. 2:**
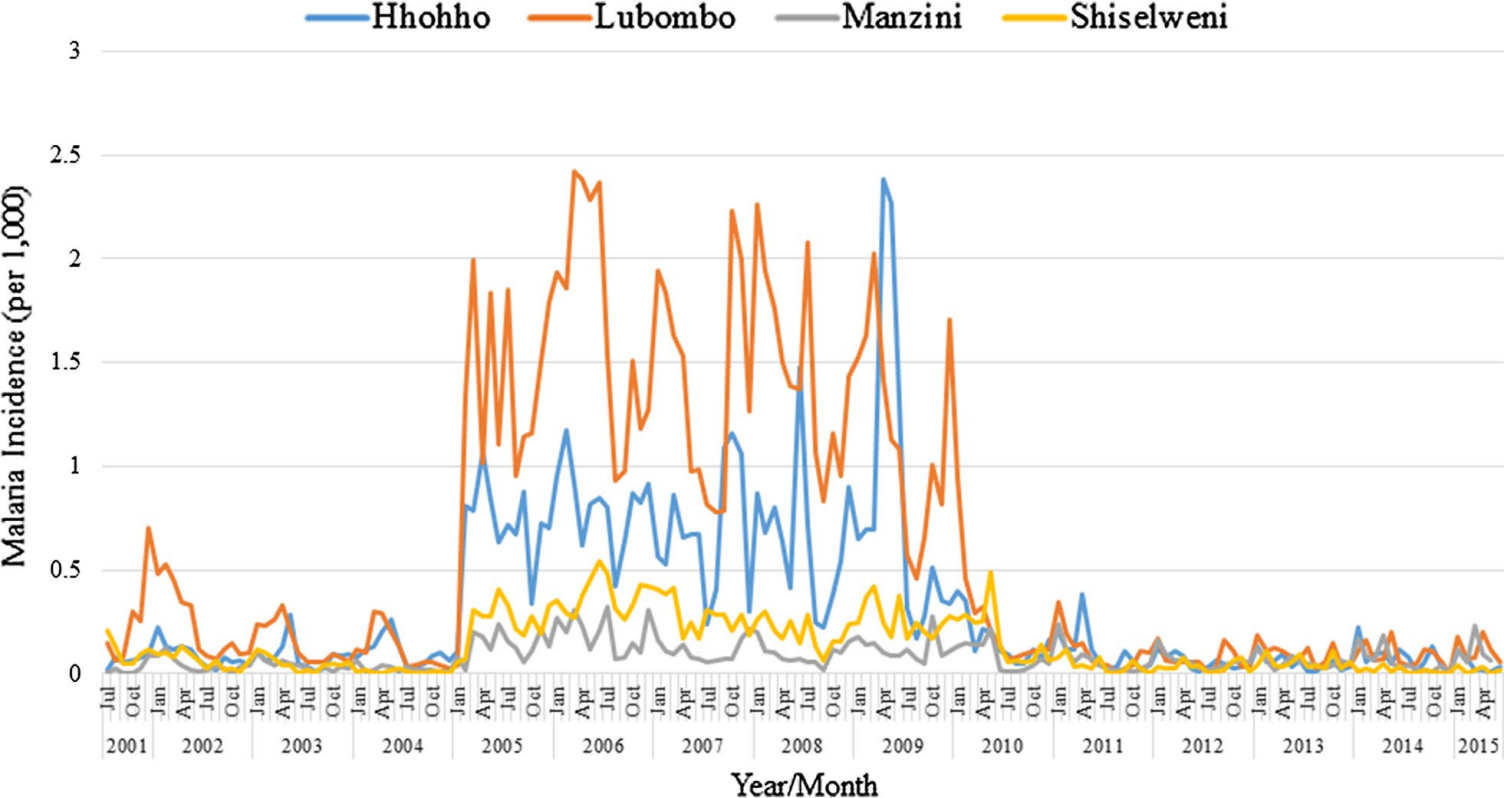
Monthly malaria incidence in the four administrative regions in Swaziland, 2001–2015

Monthly malaria incidence in Swaziland between 2001 and 2015 is shown in Fig. 2. Malaria transmission stayed below one per thousand population except for the period between 2005 and 2010. During this period, the increase in incidence was highest in the Lubombo administrative region, followed by the Hhohho, Shiselweni, and Manzini administrative regions.

Multiple climatic variables fitted by SARIMA models in each region are listed in Table 1. The analysis selected the best fitted model including parameters for autoregressive, moving average, and seasonal component of different order. The results indicate that temperature and rainfall were strongly seasonal, and were thus captured by the seasonal components (P, D, Q). However, the seasonality of malaria incidence was captured only in Lubombo and Manzini. The MEI did not show seasonality; it merely echoed its irregular characteristics.

**Table 1:** Best-fitted seasonal autoregressive integrated moving average (SARIMA) of malaria prevalence and meteorological parameters in four administrative areas in Swaziland

Variables	SARIMA (p, d, q) (P, D, Q) s	AR (1)	AR (2)	MA (1)	SAR (1)	SAR (2)	SMA (1)
Hhohho							
Malaria	(2, 1, 1)	0.610	−0.210	−0.830			
MEI	(1, 1, 0)	0.331					
TMAX	(1, 0, 0) (1, 0, 0)12	0.205			0.717		
TMIN	(1, 0, 0) (2, 0, 2) 12	0.250			0.429	0.249	
PREC	(1, 0, 0) (2, 0, 0) 12	0.209			0.078	0.079	
Lubombo							
Malaria	(1, 1, 1) (0, 0, 1) 12	0.514		−0.796	0.170		
MEI	(1, 1, 0)	0.331					
TMAX	(2, 0, 1) (2, 0, 0) 12	1.137	−0.360	−0.851	0.355	0.400	
TMIN	(0, 0, 0) (2, 0, 0) 12				0.419	0.307	
PREC	(2, 0, 1) (2, 0, 0) 12	1.031	−0.270	−0.822	0.299	0.273	
Manzini							
Malaria	(0, 1, 1) (1, 0, 1) 12			−0.682	0.885		−0.705
MEI	(1, 1, 0)	0.331					
TMAX	(1, 0, 0) (2, 1, 0) 12	0.150			−0.601	−0.286	
TMIN	(1, 0, 0) (2, 0, 0) 12	0.169			0.492	0.281	
PREC	(1, 0, 0) (2, 0, 0) 12	0.223			0.275	0.419	
Shiselweni							
Malaria	(1, 1, 1)	0.342		−0.718			
MEI	(1, 1, 0)	0.331					
TMAX	(1, 0, 0) (2, 0, 0) 12	0.232			0.394	0.426	
TMIN	(0, 0, 0) (2, 0, 0) 12				0.539	0.276	
PREC	(1, 0, 0) (2, 0, 0) 12	0.172			0.304	0.274	

*AR* autoregressive, *MA* moving average, *SAR* seasonal autoregressive, *SMA* seasonal moving average

To evaluate the importance of climate conditions in relation to malaria incidence, multivariate SARIMA models were constructed to integrate specific climate variables into malaria SARIMA models in each area (Table 2). For instance, the malaria (2,1,1) model (Table 1) was used in the Hhohho region, and other climatic variables were included in the model to evaluate climatic parameters that can better explain malaria transmission. The effect of climate variables on malaria transmission in different areas was assessed by AIC values, which would decrease if incorporating the climate variables improved the predictive power of the model of malaria transmission. In Lubombo and Hhohho, precipitation was associated with malaria transmission risk (AIC difference: −4.17 in Hhohho and −5.75 in Lubombo) (Table 2). Maximum temperature was also an important parameter in Lubombo (AIC difference: −3.46). In Manzini and Shiselweni, climate parameters did not increase the predictive performance of the malaria models, and thus probably had less of an effect on malaria transmission.

**Table 2:** Multivariate seasonal autoregressive integrated moving average (SARIMA) models of malaria incidence in four administrative areas in Swaziland

SARIMA model^a^	Coefficients	SE	AIC	AIC difference
Hhohho				
Malaria only			9.6	–
Malaria + MEI (lag = 2)	−0.067	0.049	11.67	2.07
Malaria + TMAX (lag = 0)	0.0137	0.0152	10.97	1.37
Malaria + TMIN (lag = 3)	0.0124	0.0089	12.58	2.98
Malaria + precipitation (lag = 3)	0.02	0.0066	*5.43* ^b^	−*4.17*
Lubombo				
Malaria only			92.34	–
Malaria + MEI (lag = 1)	−0.2039	0.05	89.46	−2.88
Malaria + TMAX (lag = 3)	0.0449	0.0775	88.88	−3.46
Malaria + TMIN (lag = 1)	0.0135	0.0092	92.22	−0.12
Malaria + precipitation (lag = 2)	0.0224	0.0007	*86.59* ^b^	−*5.75*
Manzini				
Malaria only			−*474.99* ^b^	–
Malaria + MEI (lag = 3)	0.0054	0.0085	−471.65	3.34
Malaria + TMAX (lag = 3)	0.7475	0.3119	−471.83	3.16
Malaria + TMIN (lag = 2)	0.0004	0.0024	−471.27	3.72
Malaria + precipitation (lag = 1)	0.0054	0.0025	−471.12	3.87
Shiselweni				
Malaria only			−*396.8* ^b^	–
Malaria + MEI (lag = 7)	0.0156	0.0139	−375.52	21.28
Malaria + TMAX (lag = 4)	0.009	0.004	−389.54	7.26
Malaria + TMIN (lag = 2)	0.0039	0.0023	−393.72	3.08
Malaria + precipitation (lag = 3)	0.006	0.0032	−390.88	5.92

^a^The lag is selected using a cross-correlation function

^b^The model with the lowest AIC value is indicated in italic type

**Fig. 3:**
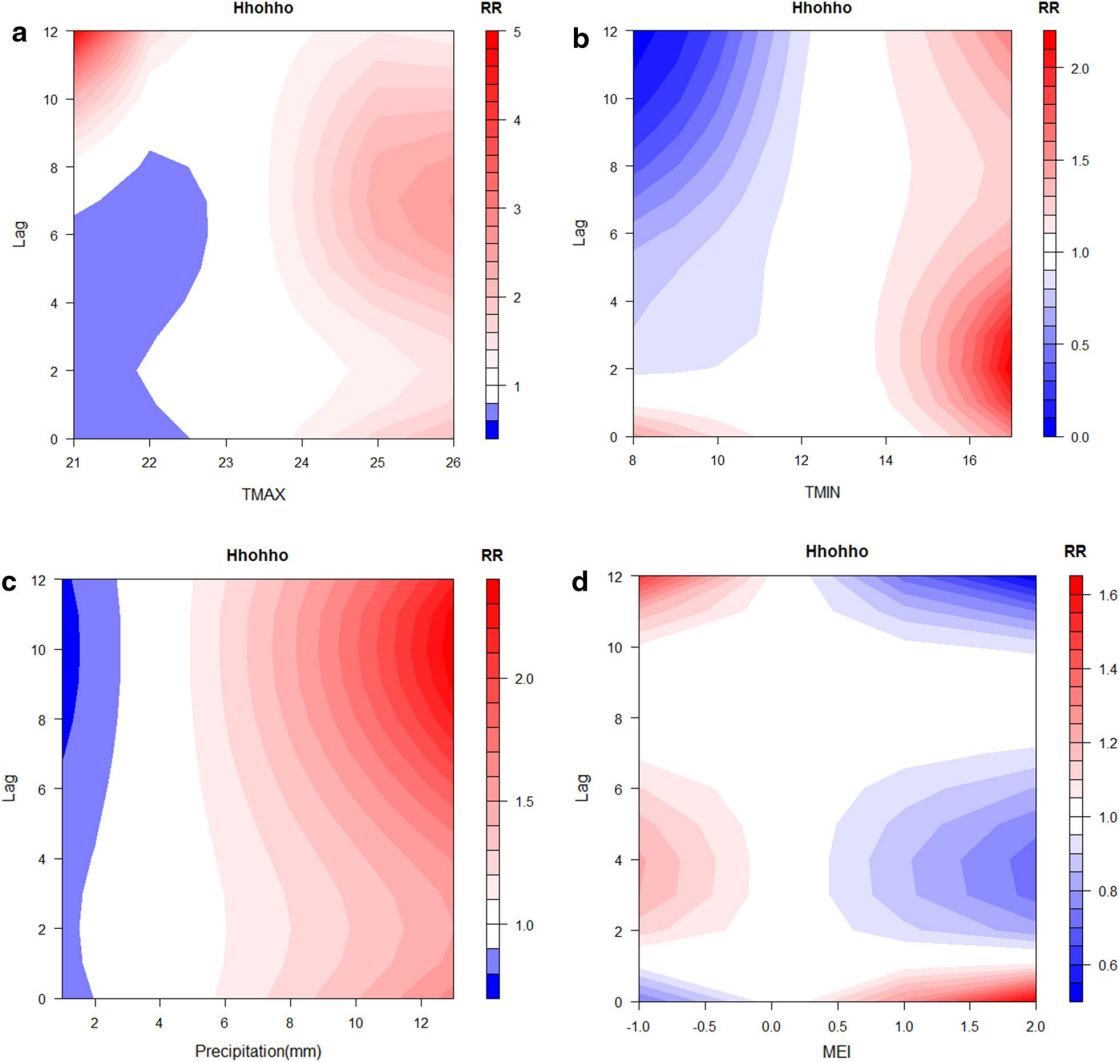
Contour plot of malaria incidence and **a** maximum temperature, **b** minimum temperature, **c** precipitation, and **d** multivariate El Niño Southern Oscillation Index (MEI) in Hhohho, 2001–2015

**Fig. 4:**
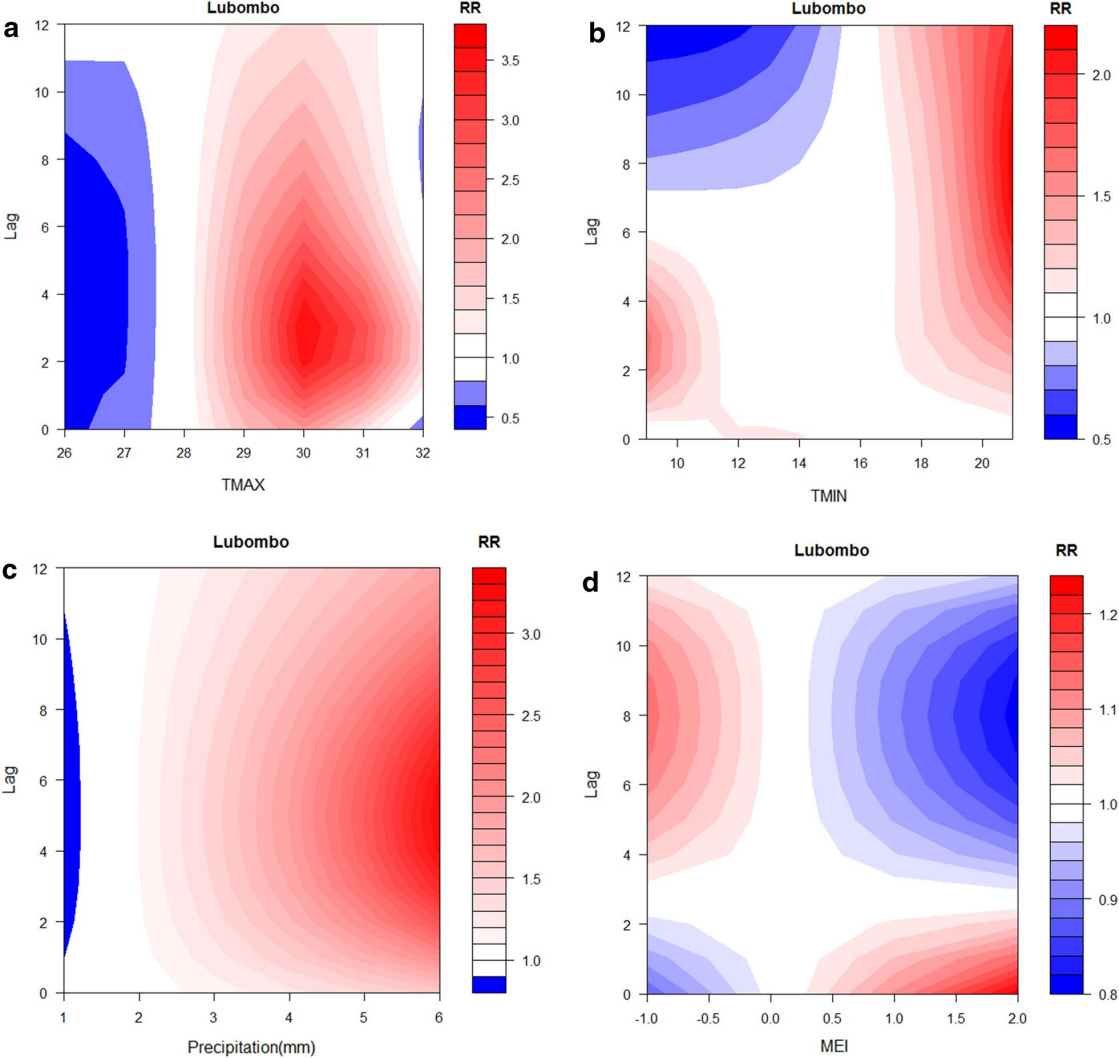
Contour plot of malaria incidence and **a** maximum temperature, **b** minimum temperature, **c** precipitation, and **d** multivariate El Niño Southern Oscillation Index (MEI) in Lubombo, 2001–2015

The DLNM approach was used to scrutinize the relationships between climate conditions and malaria transmission in Lubombo and Hhohho (Figs. 3, 4). The results showed that malaria transmission risk increased in Hhohho when the maximum temperature was above 25 °C or the minimum temperature was above 15 °C, with the effect of minimum temperature especially pronounced at a 2-month lag. Monthly precipitation above 10 in. also exhibited continuous effects, which predominated at the 6–10-month lag. In Lubombo, a maximum temperature above 30 °C increased malaria transmission risk predominantly 2 months later, as did higher rainfall (above around 5 in.) in the previous four to 6 months. The effect of the MEI was relatively weak and arbitrary in both areas (Figs. 3, 4).

## Discussion

This study is a preliminary analysis of the impact of climate variations on malaria transmission in Swaziland between 2001 and 2015. As the country approaches malaria elimination, efforts now focus on detecting all cases in Swaziland’s remaining receptive areas and preventing onward local transmission [6, 31]. The success of malaria control in Swaziland is attributable mostly to the Lubombo Spatial Development Initiative (LSDI), which began in 1999 and ended in 2011 [6]. The Mozambique, South Africa, and Swaziland (MOSASWA) cross-border malaria elimination initiative launched in 2015 seeks to renew regional efforts to accelerate malaria elimination [3]. Climate conditions are an important factor in altering disease ecology and transmission probability, not only in Swaziland, but also in neighbouring countries such as Mozambique and South Africa.

This study indicated that climate conditions were more important in the Hhohho and Lubombo administrative regions, implying that residents in these areas are at higher risk of infection when temperatures and precipitation are suitable for malaria transmission. The increased incidence in Lubombo and Hhohho between 2005 and 2010 might be associated with climate variations. Correlations among climatic parameters, mosquito development, and parasite life cycle (specifically extrinsic incubation period) have been noted elsewhere [32–34]. Dlamini et al. constructed multiple models that correlate mosquito larva abundance with 4 weeks lagged land surface temperature [35]. The non-linear patterns of temperature and precipitation relevant to malaria transmission have been discussed as well [36, 37].

The impact of regional climate phenomena on malaria transmission was also studied recently. Bouma et al. showed that El Niño conditions might have contributed to the 2016–2017 malaria outbreaks in Ethiopia [16]. Hashizume et al. also observed that the Indian Ocean Dipole (IOD) exhibits a 4-year cycle coherent with malaria seasons in the East African highlands [38]. Severe flooding due to extreme rainfall have also caused malaria outbreaks in the highland areas of western Uganda [39]. Although the multivariate El Niño Southern Oscillation Index (MEI) is not strongly associated with malaria transmission risk in Swaziland, public health authorities should nevertheless be vigilant of future climate changes and extreme local weather events that may affect ongoing malaria elimination strategies. For instance, vector control should continue along with case management, since the *Anopheles spp.* remains active in Swaziland.

Lubombo and Hhohho are two areas vulnerable to malaria transmission because of climate variations, and the two regions correspond to the cluster of locally acquired cases (Additional file 2). In contrast, most imported cases are clustered mainly near Manzini and are probably due to migration or domestic and cross-border travel. These results indicate that climate conditions might be a major driver of malaria transmission in the Lowveld ecological region in Swaziland. Accordingly, any climate-based malaria early warning system needs to be especially vigilant in the administrative regions of Hhohho and Lubombo. In addition, limited resources for disease/vector control should be deployed appropriately, noting that imported cases may trigger onward transmission within Swaziland, and that imported and locally acquired cases interact based on “malariogenic potential”, as noted by Reiner et al. [7].

Malaria transmission is influenced by multiple risk factors, and the results should be interpreted with caution or validated further, as the study has several limitations. First, malaria incidence data in the four major administrative areas are only available on a monthly basis from 2001 to 2015. Hence, it is impossible to investigate climate-vulnerable areas at a finer spatial or temporal resolution. Second, non-climatic factors were not included. In particular, there was no fine-scale data available on intervention, vector ecology, or migration and human mobility, which are also important factors driving malaria transmission. As a result, only statistical associations were established, but not causal relationships, between climate parameters and malaria. Climate parameters only partially account for malaria transmission risk. Third, it was not possible to differentiate imported and locally acquired malaria cases during the study period (2001–2015) because travel history has only been recorded since 2010. Thus, it is difficult to identify and verify the true origin of infection. This is not a major issue in long-term climatic models because climate variations affect not only Swaziland but also other neighbouring endemic countries. Fortunately, the NMCP has been actively following up both types of cases since 2010 and continues to collaborate with neighbouring countries to minimize the impact of possible misclassification. In addition, to differentiate between local and imported cases, it is worth noting that the accuracy of the rapid diagnostic test (RDT) could be poor in the low endemicity area among symptomatic patients [40]. The training of microscopy skills should be continued to maintain the efficiency of case management.

Malaria transmission dynamics can be affected by multiple factors at environmental, community, and individual levels. To strengthen the malaria early warning system in Swaziland, the current analysis should be extended. First of all, climate changes and variations are not restricted to national borders, so an analysis based exclusively on Swaziland may be inadequate to detect regional phenomena, especially since Swaziland is land-locked. Malaria transmission will not stop at the border with Mozambique or South Africa; thus, cross-border data sharing and analysis is critical. For example, a regional malaria early warning system may prove more useful, and could potentially be implemented through the MOSASWA initiative. Under such a system, an outbreak in Mozambique due to heavy rainfall might indicate an imminent spike in cases imported into Swaziland, even though there may not be unusual weather-related events in the latter.

Second, the climate effect could be buffered by different levels of herd immunity, especially for an area with high transmission intensity [12]. Although the effect might not be significant in Swaziland because of its currently low transmission status, a future regional analysis, which will include different neighbouring countries in southern Africa, should consider the interaction between climate and immunity. For example, in areas where local transmission is more persistent, it will probably be desirable to consider the impact of herd immunity on transmission, and its potential to also regulate transmission by limiting the recruitment of susceptible hosts [12, 41, 42]. Mathematical approaches could be useful in developing a dynamic malaria model to forecast transmission risk under different transmission settings within the MOSASWA area.

Third, non-climate parameters should be integrated in the future analysis, such as land cover/use, vector control, or social networks [43]. Though Swaziland is relatively small, the interaction between climate and landscape-level characteristics could be significant because of its diverse topography. With the continued financial support from and efforts by the NMCP, case management and vector control can be sustained in Swaziland. A mobile population has become the most critical challenge for malaria elimination [3]. Travel history information has been incorporated in the active investigation since 2010 by the NMCP. Social-network or contact-tracing approaches could be considered to evaluate the chains of infection and reveal the origin of infection and onward transmission [5, 44, 45]. It can provide useful information to perform a more accurate targeted control as part of MOSASWA cross-border collaboration.

## Conclusions

The impact of climate variations on malaria transmission was evaluated in Swaziland, showing that the Hhohho and Lubombo administrative regions were acutely vulnerable to climatic conditions. While Swaziland is close to a malaria-free status, the development of an early warning system could enhance the efficacy of disease control and provide a sustainable future for malaria elimination.

## Additional files

**Additional file 1.** Annual malaria incidence (black) and climate variables (red) in Swaziland, 1985–2015.

**Additional file 2.** Spatial distributions of imported and locally acquired malaria cases in Swaziland, 2010-2015. The base map was produced by kernel density estimation. (The imported cases mainly clustered in Manzini. The locally acquired cases mainly clustered in Hhohho and Lubombo).

**Additional file 3.** Correlations of climatic parameters derived from weather stations and satellite remote sensing data.
