# Supplementary figures and images for: Assessment of climate-driven variations in malaria incidence in Swaziland: toward malaria elimination
Source: Malar J. 2017 Jun 1;16:232. doi: 10.1186/s12936-017-1874-0 (PMC5455096; doi:10.1186/s12936-017-1874-0)

Additional file 1. Annual malaria incidence (black) and climate variables (red) in Swaziland, 1985-2015

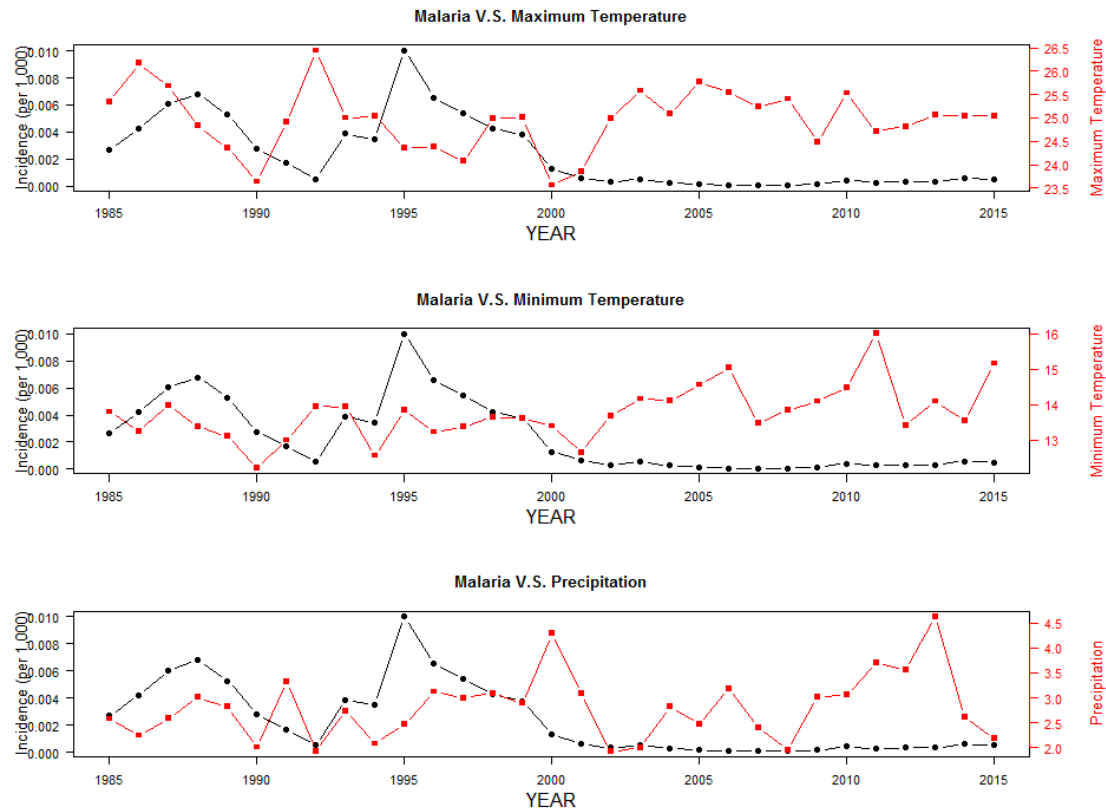

Supplement: Supplementary file 1 — Additional file 1. Annual malaria incidence (black) and climate variables (red) in Swaziland, 1985–2015. [file 12936_2017_1874_MOESM1_ESM.pdf]
