# Supplementary material for: Assessment of climate-driven variations in malaria incidence in Swaziland: toward malaria elimination
Source: Malar J. 2017 Jun 1;16:232. doi: 10.1186/s12936-017-1874-0 (PMC5455096; doi:10.1186/s12936-017-1874-0)

Additional file 2. Spatial distributions of imported and locally acquired malaria cases in Swaziland, 2010-2015. The base map was produced by kernel density estimation. (The imported cases mainly clustered in Manzini. The locally acquired cases mainly clustered in Hhohho and Lubombo.)

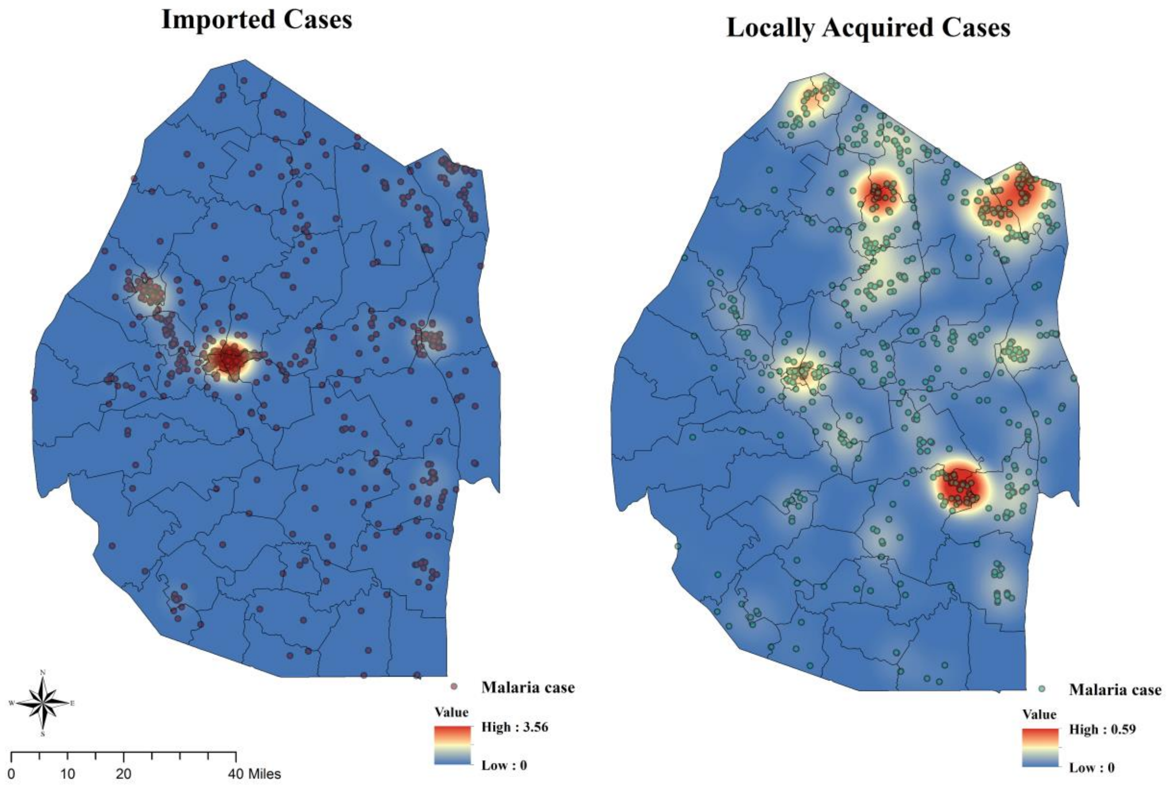

Supplement: Supplementary file 2 — Additional file 2. Spatial distributions of imported and locally acquired malaria cases in Swaziland, 2010-2015. The base map was produced by kernel density estimation. (The imported cases mainly clustered in Manzini. The locally acquired cases mainly clustered in Hhohho and Lubombo). [file 12936_2017_1874_MOESM2_ESM.pdf]
