# Supplementary material for: Assessment of climate-driven variations in malaria incidence in Swaziland: toward malaria elimination
Source: Malar J. 2017 Jun 1;16:232. doi: 10.1186/s12936-017-1874-0 (PMC5455096; doi:10.1186/s12936-017-1874-0)
Supplement: Supplementary file 3 — Additional file 3. Correlations of climatic parameters derived from weather stations and satellite remote sensing data. [file 12936_2017_1874_MOESM3_ESM.pdf]

Additional file 3. Correlations of climatic parameters derived from weather stations and satellite remote sensing data

	Hhohho			Lubombo			Manzini			Shiselweni		
	LST Day	LST Night	Rainfall	LST Day	LST Night	Rainfall	LST Day	LST Night	Rainfall	LST Day	LST Night	Rainfall
T_MAX	0.72#	-	-	0.72	-	-	0.74	-	-	0.72	-	-
T_MIN	-	0.83	-	-	0.83	-	-	0.82	-	-	0.83	-
PREC	-	-	0.82	-	-	0.82	-	-	0.92	-	-	0.78
R^2*	0.56	0.71	0.68	0.58	0.61	0.66	0.64	0.68	0.84	0.55	0.71	0.62

*R-squared statistic

#: All the correlation coefficients are statistically significant (p<0.05)

[Weather station]

T_MAX: Maximum Temperature

T_MIN: Minimum Temperature

PREC: Precipitation

[Remote sensing]

LST Day: Day time land surface temperature from MODIS

LST Night: Night time land surface temperature from MODIS

Rainfall: Precipitation from TRMM
